# Nurse-patient interactions in intensive care, transitions along the continuum of hope, and post-discharge management of chronic illness—A mixed methods narrative inquiry

**DOI:** 10.3389/fpubh.2023.1136207

**Published:** 2023-03-06

**Authors:** Gillie Gabay

**Affiliations:** School of Sciences, Achva Academic College, Arugot, Israel

**Keywords:** Bricolage, hope, intensive care, longitudinal, narrative, nurse-patient interactions, qualitative, selection mechanisms

## Introduction

In 2035, estimates are that 50% of the population will be 65 and older with a life expectancy of 100 years and an expansion of chronic illness (1, 2). When chronic illness becomes acute, patients are re-admitted to the hospital and suffer from psychological trauma (3, 4). Acute illness breaks the continuity of life, shaking the certainty in one's world. Acute illness distinguishes between life prior to the illness and life since the illness. In acute illness one re-examines life's sequence from the safe past to the shaky present through the vague, unknown perspective, into the unsettled future. Chronic illness can result in depression, anxiety, low determination to fight the disease, poor self-management of illness, and low quality of life (5, 6). Patient-nurse interactions can lead to self-management of chronic illness, but chronically ill patients report low satisfaction with experiences of nursing care (7, 8). Studies have called to promote interventions to improve nurse-patient interactions (9, 10).

Hope is one of the core psychological resources for effective coping with traumatizing illness and may facilitate self-management of illness (11).

Psychodynamic features of the theory of hope, calls to revisit hope and test nurse-patient communication that instills hope in patients facilitating self-management of chronic illness post-discharge. What place does hope occupy upon the deterioration of one's health? Hope is a psychological phenomenon, a multidimensional, dynamic attribute of individuals, encompassing possibility, active involvement, and confidence in a future outcome (12). The phenomenon of hope encompasses both conscious and unconscious aspects (13). The conscious aspect of hope is manifested in one's ability to set goals and attain them (12). The unconscious aspect of hope is manifested in one's relentless pursuit to cope with distress while deepening one's commitment to a better future. Vitality and happiness are positive emotional outcomes of hope that emerge upon one's progress toward achieving one's pursuit. These positive emotional outcomes of hope are intermixed with negative emotional outcomes such as frustration and hopelessness, when one encounters impediments on the way to achieving goals (12).

Once a physical illness develops, hope may facilitate one's coping with pain, disability, and other illness-related stressors (12). Hope motivates patients to prevent health deterioration (14). Meta-analyses on hope in chronically ill adults suggest that hope sustains life (14). While hopeful patients focused on improving clinical outcomes and recuperating, patients who lacked hope were anxious and focused on self-pity, compromising healing (15). A high level of hope when facing discouraging circumstances may stem from the belief that no situation is completely devoid of hope (16).

Levi (16) outlined a continuum of hope, with distinct profiles of hope reflecting unconscious processes of pessimistic hope, reclaimed hope, and mature hope (16). Levi defines three profiles of hope (13, 16).

1. *Pessimistic hope* has a future orientation, encompassing a desire to move forward, but it is accompanied by anxiety, sadness, and fear, making it difficult to connect to the essence of hope and its outcomes. Pessimistic hope is manifested in suspicion and skepticism toward nurses and their ability to help, requiring the nurse to contain these feelings, accept them, and be continuously present. Pessimistic hope makes it possible to deal with crises by avoiding interpersonal relationships and protecting the patient from emotional overwhelming. The inner feeling is that of the false self (17). Pessimistic hope, however, is still hope, which enables an inner mental process that may lead to receiving assistance and expressing one's true self (17). Protective regression in pessimistic hope inhibits the potential for growth that underlies the essence of hope.
2. *Reclaimed hope* is inspired by others. The nurse may “bear” the hope during the patient's hospital stay and give the patient hope. Reclaimed hope is an alternative route that allows enhancement of hope by the nurse, who is external to the self (16). The patient may unconsciously entrust his or her hope to the nurse (13, 16). The nurse may sense the hope entrusted through countertransference processes. When the relationship becomes established, the hope can be retracted from the nurse to the patient.
3. *Mature hope* is a solid personal resource. Those with mature hope contain it independently to deal with general life events and crises. In mature hope the true self of the suffering patient, which is hidden in the depths of the soul, is revealed. Hope expresses tenacity, courage, bravery, and realism, creating a potential for transformation and growth. Hope, even if it is unconscious at the beginning, when the patient becomes aware of difficulties and feels frustration on the way to achieving goals, will promote a deep inner commitment to realizing goals (13). Winnicott (17) claimed that every person is born with the capacity for hope. Patients, therefore, may expect the nurse to contain their feelings and help them reconnect with the hope concealed within them (13). Mature hope requires consistent coping when facing a grim reality. Finally, hopelessness, the lowest extremity of the hope continuum, is found in depressed patients (13). Hope is a personal resource rather than a product of external circumstances, which makes it possible to view illness as the context within which hope is explored (16). Thus, hope is not attributed to the medical condition but rather to the interpretation of the patient's narrative that directs the patient's future course. What profiles of hope do patients have in coping with acute chronic illness during lengthy hospitalizations?

While qualitative studies can broaden our knowledge of factors underlying hope in hospitalized patients, most of the existent research on hope in illness is quantitative, focusing on outcomes of low and high hope in patients (18, 19). Studies on patient experiences in acute care and on nurse-patient interactions that facilitate mature hope are scarce (20, 21). Also, although individuals are the authority regarding their own experience in a health crisis, patient reports have been disregarded (4). Since patients with lengthy hospitalizations in the ICU stressed the need for nurses to recognize their crisis and support them in times of despair (22, 23), the nurse-patient interaction may be a source of hope and may yield satisfaction with care.

Hope is, to some extent, relevant for all therapeutic relationships, which makes it possible to borrow theoretical frameworks across fields (13). Borrowing the continuum of hope from psychoanalysis, the current study explores nurse-patient dyadic interactions and transitions among hope profiles during lengthy hospital stays. This qualitative study seeks to identify interactions that inspire hope during lengthy hospital stays at the ICU from the patient's perspective and to elucidate communication that may facilitate mature hope. Since narratives convey subjective interpretation of events, allowing us to understand how encounters of patients with nurses, shape patients' experiences, this study seeks to gain insights regarding patients' experiences with data that are typically unavailable from other sources (24).

This narrative study seeks to begin closing this gap in the state-of-the-art and to identify pathways by which nurses facilitate transitions among profiles of hope and satisfaction in acute care. The explorative study responds to previous calls to elucidate communication that inspires hope in patients and enhances satisfaction during hospitalizations (20). The three research questions are (a). What profiles of hope are evident in nurse-patient dyads? (b). How does the interaction with nurses facilitate transitions from hopelessness to pessimistic hope to reclaimed hope to mature hope in patients? (c) Do transitions along the continuum of hope shape patient satisfaction with nursing care post- discharge? Insights from this narrative study may allow nurses to intervene to facilitate hope in clinical practice and enhance patient satisfaction.

## Methods

### Ethics

Following IRB approval (#0076), participants who had been hospitalized several times in the last year were recruited to participate in the study. The author informed them that they could stop the interview at any time. Participants signed an informed consent form regarding their participation and publication. Identifying details of participants, nurses, and hospitals were omitted and names were replaced by pseudonyms (25).

### Participants

Ten secular Israelis aged 66–81 participated in this study post-discharge from a 1-month acute care hospitalizations in tertiary medical centers (1,200–3,200 beds) due to life endangering cancer, heart disease, neurological disorders, or motor vehicle accidents.

### Procedure

Snowball sampling was used. Two interviews were conducted with each participant at the participant's home, extending across Israel. The first interview was conducted within the first 2 days post-discharge and the second a month later, to capture the period of negative outcomes and assess satisfaction with care (26). Each interview lasted about 2 h. Participants stressed that despite their immense physical discomfort, they wished to promote change in interactions of nurses with patients at the ICU by sharing their experience. Interviews were audiotaped, transcribed verbatim, and translated from Hebrew to English. To generate a deep, unstructured narrative, as typical in narrative interviews, one question was asked (27): “How did you arrive at the hospital and what did you experience there?” The author listened attentively, making no attempt to comment, ask, or judge what they said and attempting to send a non-verbal message of comfort as participants shared their narrative. The twenty narrative interviews provided adequate information power, as information saturation was evident from in patient experiences of interactions with nurses during their hospitalizations (28).

### Mixed methods data analysis

“Qualitative narrative studies are interpretive post-positivist inquiries, eliciting the perspectives of participants which are becoming the preferred strategy of qualitative researchers, including in nursing, to understand experiences (27). Each set of interviews was analyzed separately. Data analysis was guided by the data-driven method of selection mechanisms for narratives, applied in health regarding specific events rather than life stories (22, 29). The analysis entailed the following three steps at the participant's level, and a fourth step at the group level. In the first *step*, each transcribed interview was read as a whole unit and initial themes were identified for each participant (27). In the second *step*, each narrative was analyzed using six selection mechanisms that described what participants unconsciously chose to tell and what they chose not to tell regarding their interactions with nurses (22).

The following selection mechanisms were identified: inclusion, which refers to facts and experiences reported by the participants (e.g., everything that happened during the hospital stay); clarification, which refers to events that participants highlighted (e.g., lack of updates or no interpersonal communication with nurses); omission, which refers to events that participants viewed as irrelevant for the desired endpoint (the dynamics with one's family); silencing, which refers to events that participants perceived as conflicting with the desired outcome (e.g., sharing the experiences of other patients); flattening, which refers to the minimization of events that participants perceived as unimportant (heightened distress and fear); and attribution of appropriate meaning, which refers to the meaning that participants attributed to events that are compatible with the goal, although these may not necessarily be congruent with their original meaning (nurses' perceived attitude). In the *third step*, the endpoint of each interview regarding interactions, hope profile, and satisfaction was identified, as it emerged from the analysis. The endpoint themes involved hope and interactions with nurses during the hospitalization. In the interviews conducted at 1-month post-discharge it was important to explore whether there was a transition in participants' profile of hope and to assess the satisfaction with nursing care reported by patients. In the *fourth step*, at the group level, the Bricolage method was applied to identify common elements among experiences of participants regarding interactions with nurses, profiles of hope, satisfaction and self-management of illness (30). The Bricolage method enhances the depth of the analysis, creating new insights (30).”

### Research quality criteria

The author maintained qualitative research standards, ensuring quality, rigor, and trustworthiness (27). Also, the author outlined the aims of the study and the research questions; documented data collection methods; rendered the data analysis transparent; outlined the proper sampling method to answer the research questions in a way that enables conceptual generalizability; linked the conceptual discussion of the findings to an existing theory on hope that explains the relevance of the findings to the population of nurses; included and discussed negative experiences of participants; applied the findings to the practice of nursing; and finally, evaluated the theoretical contribution of the findings (31).

## Findings

Findings from the first interview are presented in three layers. First, the profiles of hope that were identified, guided by Levi's continuum of hope theory (13, 16). Second, wellbeing in interactions and transitions among profiles of hope were analyzed. Third, common elements among subgroups were analyzed, regarding interactions with nurses, transitions among hope profiles, and satisfaction with nursing care, applying the Bricolage method.

Findings from the second interview are presented by the profile of hope and patient responsibility to manage the illness at 1-month post-discharge.

### Phase 1: Interviews upon discharge

#### Profiles of hope

##### Hopelessness

Lack of communication for long periods of time was perceived by participants as a lack of caring and deepened their sense of hopelessness:

“I was in a wheelchair. I had never been this ill, it was very scary. I did not know what had gone wrong and what to expect. My worst fear was my next fall. What would I be doing when it happened again? I just sat there for ages. No one approached me, my head exploded with thoughts about what would happen” (Adi, 78); “When I was re-admitted again with excruciating pain, the nurse recognized me but did not ask or say a thing. They didn't care about me and pushed me away like a rag: ‘Go have a stomach, liver, and kidney ultrasound at your local clinic and then come back.' I was hypoglycemic with heart fibrillation. Five clinicians gathered around my bed. They did not talk to me. No one answered my questions. At night I had another heart attack due to an infection. I had open heart surgery” [long silence, sitting hunched over on the couch] (Yoel, 81).

##### Pessimistic hope

Participants described negative emotions due to interactions with nurses:

“The nurse came by to see me but didn't notice me at all. She hardly spoke to me. I had excess fluid in my head that pressured my brain and caused a cognitive regression, loss of memory, and walking disorder. I was scared to death when I was told of the planned procedure [silence]. For them it was technical, for me… I felt intense cold throughout my body” (Koby, 74); “They did not fight for me. They were very technocratic. Basically, they did their job and that's it. They don't really care about me. If my fifth hospitalization this year taught me anything, it's that the hospital is a huge factory, and I am only a kettle that needs fixing. No one talked to me. Not a nurse, not a social worker or a psychologist” [silent] (Ella, 66).

##### Reclaimed hope

When participants were processing the crisis and felt in good hands this may have allowed them to rely on the nurses to take their hope and hold on to it, in their uncertain situation:

“I kept falling, my legs would not hold me. The nurse said, ‘the doctor read your CAT scan and there are definitely deficiencies, but the doctor will hold a consultation and let you know about surgery.' I felt in good hands. Despite my objection to surgery, I decided that if I needed surgery to come out of this, then surgery it is” (Moshe, 72).

Martin attributed his hope for recover to the encouraging communication with the hopeful nurse:

“One day the nurse announced: ‘Today we are standing'. She sensed my fear and said, ‘Don't worry, I will hold you if you fall. I checked your leg strength – you should be able to stand up'. She was tiny. I was afraid of falling, but she held me. Eventually I succeeded. It felt amazing” (Martin, 68).

##### Mature hope

“It was very late at night. The nurse said 'You are in the Intensive Care Unit (ICU) but we hope it will not spread. It is very unpleasant, but we hope you will overcome this'. I felt very close to him. He kept talking and instilled so much hope in me. After a few days, I felt that I would be okay” (Shaul, 69).

#### Interaction with nurses and transitions from hopelessness to pessimistic hope to reclaimed hope to mature hope

##### From hopelessness to pessimistic hope

When nurses shared their hope and expressed their professional view regarding rehabilitation, they facilitated the development of hope in participants.

Nurit had no desire to have surgery and shared how the nurse's fresh perspective had helped her accept her new grim reality and agree to have yet another surgery:

“I had no resources to deal with the news. I was on my way to surgery for the fifth time this year. It was too much, too much. I didn't want any hospital, any physician, any nurse or surgery. When I shared my inability to accept my new medical reality the nurse had an interesting expression. She was quiet for a few minutes. Then she looked at me and said: ‘It is challenging, yes. There is nothing simple about this. But do you know how many people would change places with you? How many would want to detect their cancer early on, remove it, and move on with life?”' (Nurit, 79).

The nurse held on to hope for Nurit, who was shocked by the grim news that the cancer had spread. In time, Nurit developed independent mature hope:

“My body is traumatized, due to the surgery. Recognizing my limitations is part of the healing process. I am functioning on a lower level, less capable, have no strength or energy. I'm at the bottom of the climbing wall of my life. I need to climb up again and again. I need a lot of physical and mental strength to deal with such a transition. But who wants to stay down there? No one! I sure don't. I'm lucky that I can continue and play the ‘music' of rehabilitation” (Nurit, 79).

##### From pessimistic hope to reclaimed to mature hope

When nurses allowed participants to share their emotional experiences, this facilitated a positive change in their emotions and attitudes.

Eli transitioned from reclaimed hope to mature hope through the communicative behavior of the nurse, which made a huge difference for him:

“‘You are not dying on me', she said. ‘You will live'. She hugged me. At that moment, I had no doubt that I would remain alive [tears]. Patients whose nurse kept their spirits up persevered, patients who had an easy condition but were depressed died because their disease spread. I think the biggest difference among doctors and nurses is their message to the patient. I think that 50% of the treatment is one's hope that they will live. It encouraged my determination to heal [quiet]. I owe her my life” (Eli, 68).

Koby added his similar experience, stressing the impact of the interaction with the nurse on processing his trauma while she held on to hope for him:

“I was so sad. Then I felt afraid. Friends and colleagues slowly drift away from you when you are that sick. The nurse would stay with me to talk about my fear of missing the weddings of our children and not knowing my grandkids. I was devastated. She would come with coffee and chocolate, and I would share what I could not share with anyone in the world. She made a huge difference for me. I was lucky” (Koby, 68).

#### Bricolage

Two common themes distinguished between the subgroups. One group of participants lacked hope both throughout their hospital stay and a month post-discharge, expressing anxiety and dissatisfaction with care. Ella, Adi, Roni, and Yoel remained devoid of hope. Yoel felt that he had been better off before the surgery, as the last operation had left him disabled:

“I was there so many times this year. They already know me by name. The experience was horrific. Hallelujah! I am home. I hope I stay home. I came out of there in a wheelchair, nothing will be the same. I will not be readmitted” (Yoel, 81) [Yoel refused to be readmitted and died 8 months post discharge].

The second group transitioned from hopelessness to pessimistic hope to reclaimed hope and to mature hope, expressing gratitude toward the nurses and satisfaction with the nursing care. Martin, Moshe, Shaul, Nurit, and Michelle had, in their view, exceptional experiences and transitioned across profiles of hope.

*Common themes in the transition from pessimistic to reclaimed hope and to mature hope Four* participants (Eli, Michelle, Martin, Moshe) experienced interactions that instilled in them hope and directed them to seek information, reappraise their resources, and believe in their rehabilitation. They were encouraged to ask the nurse questions to improve their functional limitations. Participants treated by a nurse who reflected hope transitioned to mature hope. Thus, Martin shared his transition from reclaimed to mature hope:

“You start off from the worst place, you don't know anything, you don't feel your legs, you cannot stand. Suddenly, you wake up one morning with no motor senses. You just slowly assimilate that you have no control. We can only do the best we can. That's it. If anything happens, you cope with it. I understand that I have no choice but to manage myself” (Martin, 68).

### Phase II: Interviews at 1-month post-discharge

#### Hope and responsibility for post-discharge for illness management

Participants who lacked hope upon discharge remained devoid of hope 1-month post- discharge. They expressed a fear of hospitals, disappointment with the nurses, and dissatisfaction with care. Ella elaborated on her experience and how instead of empowering her, the interaction made her feel diminutive. Participants were traumatized by insensitive communication by nurses. Ella, Yoel, Adi, and Koby, who felt a lack of hope upon discharge and a month post-discharge, accepted their functional impairment, did not feel responsible for rehabilitation and illness management and did not look forward to the future. Ella shared her point of view:

“It's not enough to know medicine if you don't understand the human soul. Nurses are technicians, they have forgotten to communicate and to be healers. They don't know how to listen; they no longer hear anything. I am disabled but if they would at least communicate with me it could have been different. I am left with the sickness, and death is approaching” (Ella, 66).

Koby felt a lack of hope and was determined to refuse surgeries in the future:

“They were condescending and did not care about me. I decided that whatever happens to me, I would not go through any more surgeries. I can hardly walk since the operation. I am not active. People should stay away from hospitals” (Koby, 74).

Participants who described hope upon discharge felt hope, gratitude, and satisfaction with the nursing care. Martin described his determination to walk again:

“I told myself, ‘No matter how long it takes, you will walk again!' Every time some negative thought surfaces like 'you will never walk again' – I shove it away. It's tough but I am encouraged. Every day I force myself to take more steps than the day before, regardless of the pain or how long it takes” (Martin, 68).

Eli, who expressed mature hope upon discharge, displayed hope and commitment for rehabilitation a month later:

“I am slowly recovering and getting back to a blessed routine. It's complex, but I have no complaints, I am lucky and so thankful. Every day I feel privileged to be alive” (Eli, 68).

Michelle expressed continued mature hope at 1-month post-discharge and gratitude after four readmissions in a single year:

“In this whole journey all we have is this moment, the here-and-now. When I reflect on the hospitalization, I remember the exceptional dedication of my nurses during the four times I was hospitalized this year. All of them were so sensitive in their communication with me. I remember the entangled, intertwined roads of my journey. A ton of hours filled with my worries and their listening, encouragement, and support. Their beneficial presence undoubtedly encouraged me to return four times to the operating table for complex surgery. Our presence is a gift. We really only have the current moment” (Michelle, 74).

**Figure 1:**
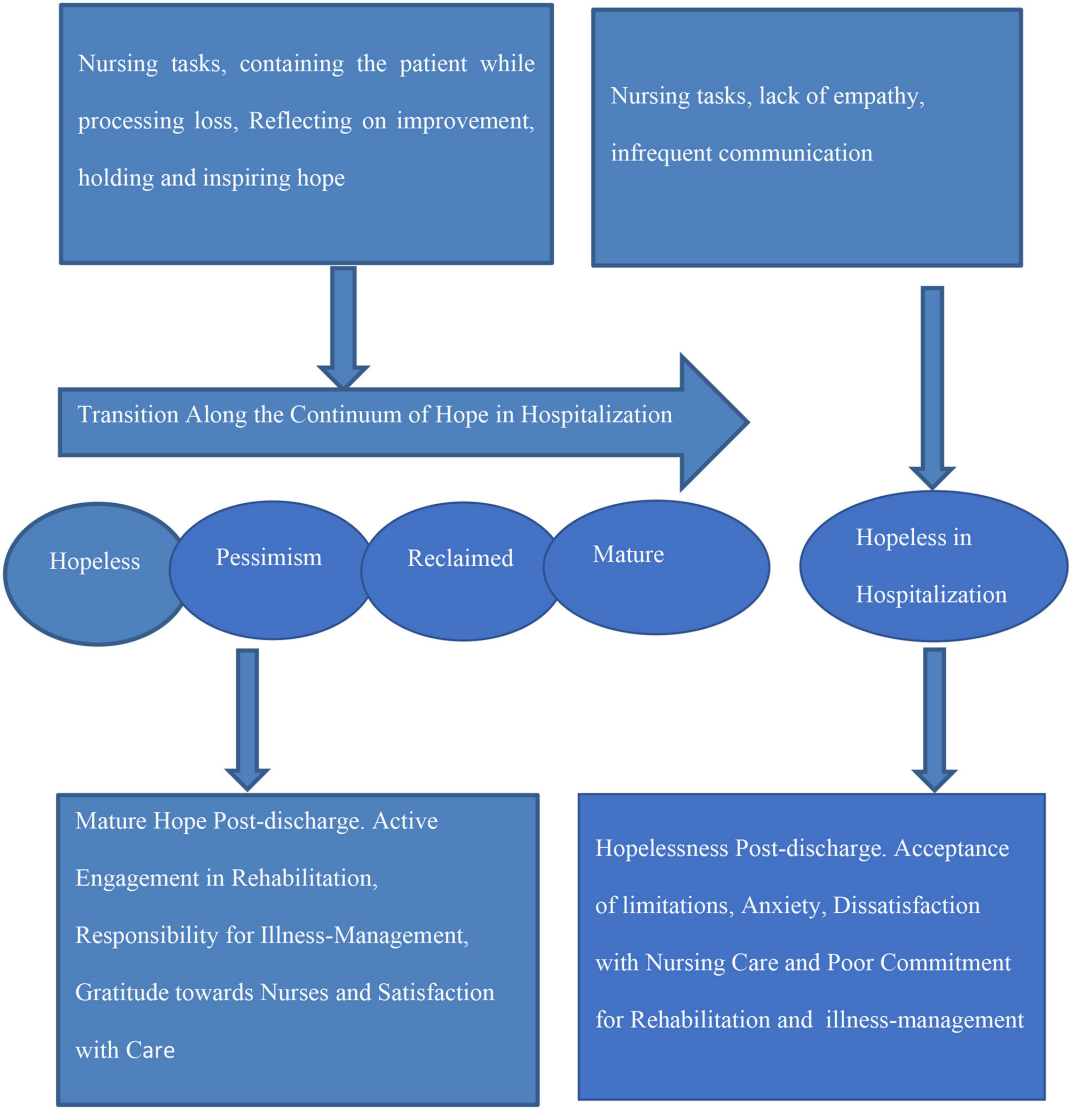
Work of hope in nurse-patient interactions in hospitalized patients and post-discharge satisfaction with nursing care.

To sum, participants whose nurses interacted with them by holding eye-level conversations, containing their negative feelings, discussing the potential for improvement, and holding on to hope for them, allowed the participants to process their negative emotions and reclaim their hope. While the participants processed their emotions, the nurses held on to hope in their stead. Nurses facilitated the development of mature hope in participants, who reported that the nurses had instilled hope in them and expressed satisfaction with the care. Participants who felt no hope upon discharge, lacked hope and were dissatisfied with care at 1-month post-discharge as well. These participants were determined to resist future readmission to the hospital. Figure 1 presents the research findings.

## Discussion

This study voices the narratives of in-patients begins to close the gap in the state of the art regarding how nurse-patient interactions may facilitate psychodynamic hope in lengthy hospitalizations and post-discharge illness management. Borrowing the hope continuum from psychoanalysis, this study explored patient interactions with nurses, patient transitions across profiles of hope during lengthy hospitalizations in intensive care, and patient self-management of illness. This mixed-methods qualitative study has several contributions. Theoretically, the study extends the knowledge on psychodynamic hope in nurse-patient interactions in hospitalizations and links it to patient self-management of illness. Insights from this study broaden our understanding of the mechanisms of psychodynamic hope and reaffirm that hope is essential for patients and that nurses are in a unique position to improve patient satisfaction in lengthy hospitalizations by building nurse–patient relationships that instill hope.

Methodologically, this study is based on the patient perspective. This study analyzed narratives by the selection mechanisms method at the individual level to reveal hidden layers in nurse-patient interactions and using the Bricolage method at the group level, it demonstrated that qualitative narrative research can produce insights even shortly after a health crisis. Practice recommendations of this study elucidate interactions that facilitate movement from hopelessness to hope during hospitalization, and self-management of illness post-discharge.

## Profiles of hope among patients in intensive care

Findings highlight the nurse-patient interaction as a source of hope and of satisfaction with care. Patients in intensive care may experience a painful separation from hope, which then becomes conspicuous by its absence as the patient feels pain, fear, overwhelming symptoms, and a weakening of the self (22). The patient needs to experience acceptance and no judgementalism which may facilitate psychological safety and self-reflection (22). When patients are frustrated, they need nurses to contain their frustration, anger, despair, and hopelessness, until they are able to generate hope within themselves (13, 16). Hope may not necessarily originate from the patient who is experiencing loss and trauma, may be inspired by nurses, who may serve as a bridge to hope. The interaction with the nurse may allow patients to reclaim their hope from the nurse who functions as a source for hope and develop mature hope (13).

### Interactions with nurses and the process of transitioning along profiles of hope

Fostering hope in situations of loss requires reconciling the patient's despair with the nurse's attitude of potential change, enabling patients to contain the despair while developing hope Despair is shared with the nurse, who “sees” the patient through empathic attunement and through interpretation and sense making, thereby meeting the patient's need for containment (18). The patient may encounter self-alienation and aloneness due to processing the loss, but at the same time may contact and share the distress with the nurse (22). In these moments the nurse may "hold on to” the patient's hope, and when the patient is ready, pass it back to the patient with realistic anticipation and mature hope (16). When the patient accepts vulnerability and copes with the hospitalization as a challenge that can be overcome, there is a potential for mature hope (13). Hope and hopelessness are important determinants of emotional wellbeing, increasing comfort and life satisfaction (32). Findings support studies on the powerfulness of hope (14, 15). The Nurse-patient interaction may help the patient move through the process from regression to dependence, enabling a connection with the nurse and satisfaction with care (33, 34).

### Hope during the hospitalization and post-discharge

Without transitioning along the continuum of hope, participants felt no hope both throughout their hospitalization and a month post-discharge. Where there was no hope, there was no post-discharge self-management of illness among participants. Since in the dyadic relationship, nurses may also experience an overwhelming sense of their own helplessness, inability to “do something” to revive the treatment, inability to maintain hope, and perhaps identifying with patients' sense of hopelessness, it is complex process of bringing together the nurse, who is willing to help unconditionally, and the patient who is not completely certain of her or his needs (22). To set in hopes for patients, it's essential for nurses to distinguish between patients' gratification for the relationship with the nurse and patients' underlying frustration with limitations in functionality. Careful reassurance of the nurse-patient relationship and sensitivity of the nurse may facilitate independent hope in patients even when they are facing a grim reality making hope a phenomenon that is inextricably intertwined with the dynamics of the relationship in the here-and-now of hospitalizations.

Compatible with a recent study, hope should be fostered by providing information to help patients develop an understanding of the motional difficulties and encourage their active role in their self-care (32). In contrast to previous studies, however, even hopeful patients need a space of self-alienation from friends and relatives when they were hospitalized as they are unable to share their feelings with others (12, 22). Exploring the importance of the nurse-patient interaction, the findings are compatible with a study which found that nurse–patient interactions are a source of hope in nursing homes (33). Nurse-patient interactions that inspire hope, may empower nurses through granting greater meaningfulness to building relationships with patients (35).

## Implications for practice

To increase patient satisfaction with nursing care throughout the hospitalization and post-discharge, nurse-patient interactions should instill hope in patients who face loss and hopelessness in lengthy hospital experiences. Relationships entail an active presence of nurses as providing comfort and hope, which translate into higher involvement with patients in their care. The findings call upon nurses to first acknowledge patients' feelings regarding their crisis and to be aware of the importance of embracing patients' despair and interacting in a way that leads patients to mobilize courage and develop hope. Nurses may facilitate the development of hope by being sensitive toward the unique psychological needs of patients, and by assessing patients' ego strength and fragility (18). The nurse may honor the patient's despair through empathy on one hand and hold the conviction that the patient can and should progress on the other. Training may target (a) the art of containing the emotional needs of patients while working in a chaotic work environment of intensive care; (b) shifting from providing clinical care to integrating clinical nursing care with building positive relationships with patients so they can lean on nurses until they develop hope independently; (c) since patients may perceive the nurse with both hope and suspicion, it is imperative that training also targets skills of attentiveness, empathy, and reflection. It is extremely important to incorporate work with hope in nursing care. A vision of hope as a source for better recovery and patient satisfaction should be incorporated into all treatment models.

## Conclusions

Hope in hospitalizations is psychodynamic and essential for patients. Nurses are in a unique position to improve patient satisfaction in lengthy hospitalizations by building nurse–patient relationships that instill hope affecting post-discharge self-management of illness.

## Limitations and directions for future research

The findings link nurse-patient interactions in acute care to hope and satisfaction with care. While the role of the patient-nurse interaction is critical, one must bear in mind that narratives and the interpretation of events are affected by the personality, construction of reality, life experience, culture, and emotional and cognitive state of each participant. These limitations call for further research which may explore both hope and satisfaction with nursing care among hospitalized patients in a quantitative study with a large sample. Future studies may also explore the experiences of nurses in the context of instilling hope through interactions with hospitalized patients.

## Author contributions

The author confirms being the sole contributor of this work and has approved it for publication.
